# Formative Study to Inform a Physical Activity Intervention Targeted to Rural Men in the United States

**DOI:** 10.1002/hsr2.70485

**Published:** 2025-02-19

**Authors:** Jacob Gallagher, Emine O. Bayman, Lisa A. Cadmus‐Bertram, Nathaniel D. M. Jenkins, Amy Pearlman, Kara M. Whitaker, Lucas J. Carr

**Affiliations:** ^1^ Department of Health and Human Physiology University of Iowa Iowa City Iowa USA; ^2^ Department of Health and Kinesiology Iowa State University Ames Iowa USA; ^3^ Departments of Biostatistics and Anesthesia University of Iowa Iowa City Iowa USA; ^4^ Department of Kinesiology University of Wisconsin Madison Wisconsin USA; ^5^ Prime Institute Fort Lauderdale Florida USA; ^6^ Department of Epidemiology University of Iowa Iowa City Iowa USA

**Keywords:** health promotion, intervention development, men's health, needs assessment, ORBIT model, physical activity

## 1 Introduction

American rural men have a higher risk of inactivity‐related disease and premature mortality compared to rural women and urban men [1, 2, 3]. However, only 27.6% of rural men meet the physical activity guidelines [4]. Our previous study found that 80% of rural American men were interested in a physical activity intervention [5], yet rural men make up a small proportion (< 15%) of people in physical activity interventions [6].

Preliminary evidence from Australia suggest that rural men may be interested in tailored interventions [7]. While rare, physical activity interventions specifically tailored to men have been effective at recruiting and retaining men; however, these past studies have been targeted primarily to urban men outside of the United States [8, 9].

The Obesity‐Related Behavior Intervention Trials (ORBIT) model [10] was developed to develop behavioral interventions efficiently, effectively, and targeted to the needs of specific populations, and uses similar terminology to testing new pharmaceutics. During Phase Ia (define), the basic elements of the intervention are defined, including the behavior change theory used, appropriate population, and barriers to be addressed. In Phase Ib (refine) of the ORBIT model, the mode of delivery and intervention features need to be identified.

Our prior research met the benchmark in Phase Ia [5], but additional evidence is needed to meet the benchmarks in Phase Ib of the ORBIT model. The aim of this study is to reach those milestones by identifying: (1) potential psychosocial constructs to targets, (2) behavior change techniques, (3) channels of delivery (how information is delivered and by whom), and (4) a specific focus of the program.

## 2 Methods

### 2.1 Participants

Rural men (447 total invites), who completed our initial needs assessment [5], were recontacted via Amazon's Mechanical Turk. To be included, participants needed to self‐identify as male, be over 18 years old, and live in the United States in a town of less than 2500 people or outside of a town. All procedures were approved by the Institutional Review Board at the University of Iowa and were performed in accordance with the Declaration of Helsinki. All participants provided informed consent before participating in the study.

### 2.2 Measures

#### 2.2.1 Psychosocial Constructs

Participants completed an online survey (see Rhodes, 2021 [11]) that measured psychosocial constructs related to physical activity behavior (attitudes, capability, opportunity, behavior regulation, habit, and identity) that align with the multi‐process action control theory, a meta‐theory that draws from multiple existing psychosocial theories related to physical activity [12, 13].

#### 2.2.2 Behavior Change Techniques

Participants were asked to select behavior change techniques previously identified in Michie's behavior change taxonomy [14] that they would find helpful in a physical activity program (e.g., demonstrations, encouragement).

#### 2.2.3 Channels of Delivery

Participants were asked to rank their preference for various channels of receiving information (e.g., website, app.) and the mode of presentation (e.g., presenter characteristics).

#### 2.2.4 Focus of the Program

Lastly, participants selected preferred program focuses including modes of exercise (e.g., strength, cardio) and targeted health benefits (e.g., physical fitness, mental health).

### 2.3 Data Analysis

Data were summarized by descriptive statistics, including frequencies, means, standard deviation, and percentages using R.

## 3 Results

Men completing the survey (*n* = 131) were mostly White (89.5%), non‐Hispanic (93.9%), college educated (84.2%), married (57.0%), and full‐time workers (62.6%). Ages ranged from 18 to 79 years (median: 39 years old). Using IP address, participants were from the Midwest (*n* = 43), Northeast (*n* = 21), South (*n* = 38), and West (*n* = 21) census bureau regions (8 without an identifiable region) in the United States.

Participants reported high levels of instrumental attitudes (Mean ± standard deviation: 16.1 ± 2.0 out of 18), perceived capability (15.1 ± 3.1 out of 18), perceived opportunity (14.9 ± 3.4 out of 18), and affective attitude (13.2 ± 3.6 out of 18), but reported lower levels of behavioral regulation (17.4 ± 8.8 scored out of 36), habit formation (13.5 ± 7.0 out of 24), and physical activity identity (16.1 ± 5.8 out of 24). Rural men reported exercise demonstrations as the top choice (selected by 74.0%) of behavior change techniques (Table 1).

**Table 1:** Behavior change techniques chosen by rural men.

Behavior change technique	% of respondents selecting as a helpful feature
Demonstrations and instructions on how to exercise	75.6%
Benefits of physical activity (including weight loss and management, disease prevention, healthy aging)	68.9%
Encouragement to be physically active	67.8%
Biofeedback on your physical activity (e.g., steps per day)	67.8%
Techniques to help you stay active (e.g., goal setting, developing habits, tips/tricks)	65.6%
Tools/techniques to manage time	54.4%
Stress management techniques	53.3%
Info on how to overcome barriers that are preventing you from being active	51.1%
Personalized feedback on your physical activity goals	42.2%
Info on how active I should be	41.1%
Info on how to set physical activity goals	35.6%
Costs of different physical activities	33.3%
Opportunities to connect with others through an online community	32.2%
Risks of being inactive	30.0%
Reminders throughout the day to be physically active	30.0%
How other men view exercise	22.2%
Comparison to other men's physical activity (e.g., leaderboards. competitions)	20.0%
Opportunities to speak with a health coach	20.0%

More than half of all participants (55.7%) reported a preference for a male presenter in exercise demonstration videos while a third (32.8%) had no preference. A fitness professional (e.g., exercise physiologist) was the most preferred person to demonstrate an exercise (Mean ± SD: 4.3 ± 0.9 on a scale of 1‐Dislike a great deal to 5‐like a great deal), followed by a peer (3.6 ± 2.0), medical professional (3.2 ± 1.2), spokesperson (e.g., celebrity; 2.9 ± 1.3), and local leaders (2.7 ± 1.4).

**Figure 1:**
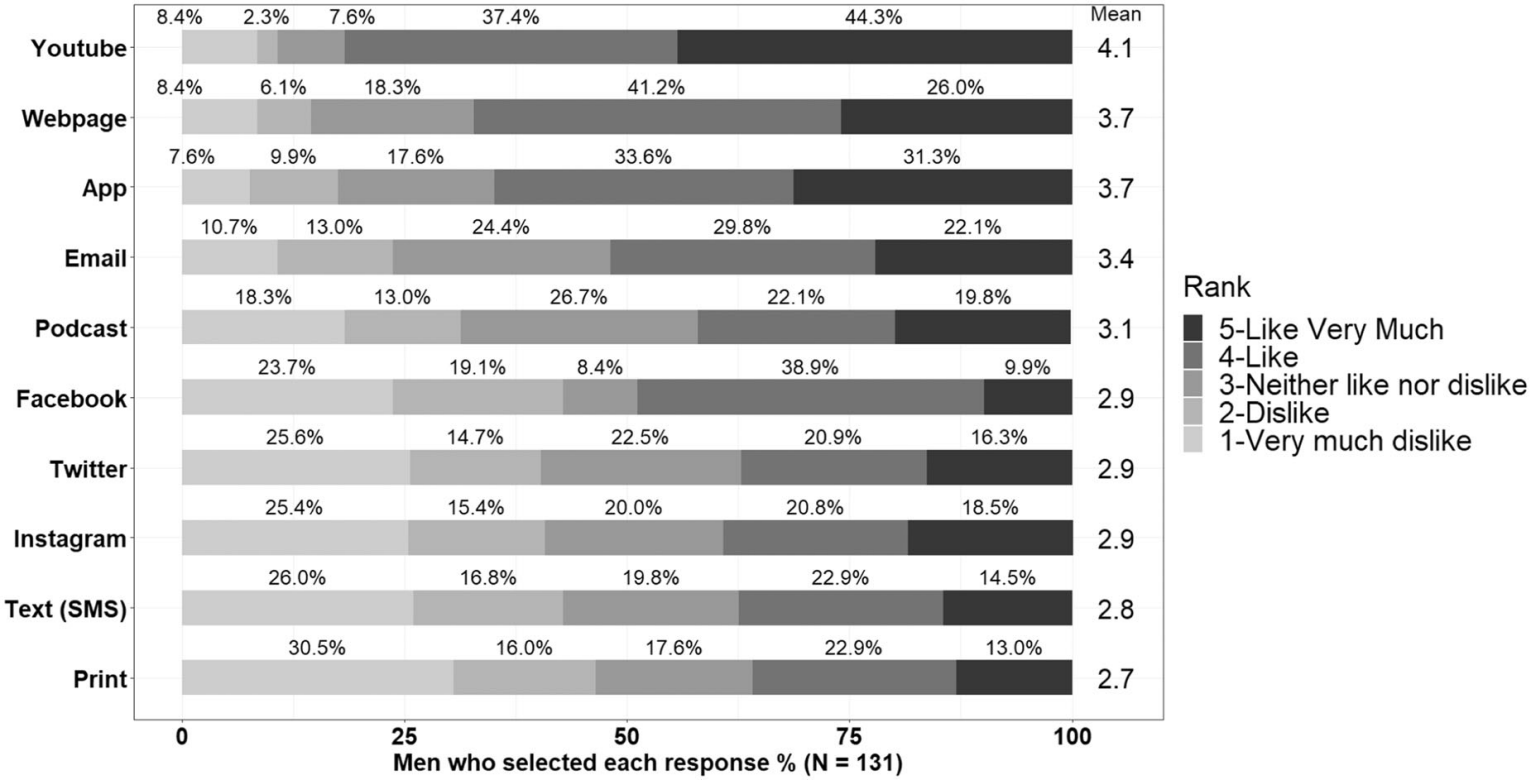
The percent of men selecting like or dislike for modes of delivery with the mean response represented on the right‐side column.

YouTube videos (Mean ± SD: 4.1 ± 1.2 on a scale of 1‐dislike very much to 5‐like very much) was the most preferred method of receiving educational information (Figure 1). When asked who should deliver the information, rural men's top choices were fitness professionals (Mean ± SD: 4.2 ± 0.9 on a scale of 1‐Dislike a great deal to 5‐like a great deal) and medical professionals (3.8 ± 1.0). For delivery of educational materials, 51.9% preferred a male, while 38.0% had no preference.

Rural men reported wanting information on how physical activity can improve “physical fitness” (selected by 84.0%), “mental health” (69.5%), and “sleep” (58.0%). Most men (68.7%) reported they wanted a program that focuses on self‐improvement compared to 46.6% who selected a leisurely program and 38.9% who selected a competitive program. The modalities of exercise men most preferred were strength (80.9%), cardio (69.5%), and balance (67.2%).

## 4 Discussion

Our findings support the use of YouTube videos of a male fitness professional demonstrating rural men's preferred exercises, strength, aerobic (cardio), and balance with an emphasis on self‐improvement. Previous research suggests such videos should use simple, straightforward language, and humor when appropriate [9].

American rural men may view purposeful physical activity (e.g., manual labor) as more socially acceptable and are motivated to slow aging and maintain strength [15]. In line with this, we found a slight preference for strength training and a focus on self‐improvement instead of leisurely focus. In tailored men's health research, it may be important to tailor to masculine norms to draw men in to the program, but once in the program, these norms might be slightly modifiable [16]. For example, advertising a strength program may get men through the door, but once they “buy in,” they may be convinced to do more aerobic activity or other health behaviors (e.g., eat healthier).

An intervention that promotes behavioral regulation, such as providing a tracking device, may be best suited for rural men, as attitude and capability were both reported near maximum values. In a previous study using a weight loss app, rural men found behavior regulation an acceptable and helpful feature [17]. However, the efficacy of app was limited [18], which suggest that not only does the psychosocial elements need to be targeted but other features (channel of delivery, focus of program) may need to be tailored to promote efficacy.

Our sample was more educated and had more White participants than the national average for rural communities [19]. To improve representation of education status of rural men, alternative recruitment methods are warranted, such as ads (radio, newspapers) and through existing networks (e.g., extension outreach, Farm Bureau). This may be particularly important to how information is presented and received by those with less education. A strength of this study is the use of a nationwide survey, allowing for more generalizability in the United States than a geographic specific study. Similarly, there was a wide age range represented.

## 5 Conclusion

These findings provide a basis for a Phase IIa proof‐of‐concept study to test the initial feasibility, acceptability, efficacy of an intervention that tailors to rural men by focusing on demonstrations of physical activity and behavioral regulation.

## Author Contributions

**Jacob Gallagher:** conceptualization, investigation, funding acquisition, writing – original draft, methodology, validation, visualization, writing – review and editing, software, formal analysis, project administration, data curation, supervision, resources. **Emine O. Bayman:** writing – review and editing, formal analysis, conceptualization. **Lisa A. Cadmus‐Bertram:** writing – review and editing, methodology, conceptualization. **Nathaniel D. M. Jenkins:** writing – review and editing, formal analysis, conceptualization. **Amy Pearlman:** conceptualization, writing – review and editing. **Kara M. Whitaker:** conceptualization, writing – review and editing. **Lucas J. Carr:** conceptualization, funding acquisition, writing – review and editing, supervision, project administration.

## Conflicts of Interest

The authors declare no conflicts of interest.

## Transparency Statement

The lead author Jacob Gallagher affirms that this manuscript is an honest, accurate, and transparent account of the study being reported; that no important aspects of the study have been omitted; and that any discrepancies from the study as planned (and, if relevant, registered) have been explained.

## Data Availability

The data that support the findings of this study are available from the corresponding author upon reasonable request.
